# Distinct tumor microenvironments of lytic and blastic bone metastases in prostate cancer patients

**DOI:** 10.1186/s40425-019-0753-3

**Published:** 2019-11-08

**Authors:** Claire L. Ihle, Meredith D. Provera, Desiree M. Straign, E. Erin Smith, Susan M. Edgerton, Adrie Van Bokhoven, M. Scott Lucia, Philip Owens

**Affiliations:** 1 Cancer Biology, University of Colorado Anschutz Medical Campus, Aurora, CO 80045 USA; 2 Department of Pathology, University of Colorado Anschutz Medical Campus, Aurora, CO 80045 USA; 3 Research Service, Department of Veterans Affairs, Eastern Colorado Health Care System, Aurora, CO 80045 USA

**Keywords:** Prostate, Bone metastases, Tumor microenvironment, Digital spatial profiling

## Abstract

The most common metastatic lesions of prostate cancer are in bone and can be classified into three distinct pathology subtypes: lytic, blastic, and an indeterminate mixture of both. We investigated a cohort of decalcified formalin-fixed and paraffin-embedded (FFPE) patient specimens from the bone that contained metastatic prostate cancer with lytic or blastic features. These tissue sections were utilized for immunohistochemistry (IHC) staining, isolation of RNA for gene expression, and Digital Spatial Profiling (DSP) of changes in both the tumor and microenvironment. A diverse set of unique immune cell populations and signaling pathways to both lytic and blastic types of prostate cancer metastases were present. In blastic lesions immune cells were enriched for pSTAT3 and components of the JAK-STAT pathway. In lytic-type lesions, immune cells were enriched for pAKT activity and components of the PI3K-AKT pathway. Enrichment for immune checkpoints including PD-L1, B7-H4, OX40L, and IDO-1 were identified in blastic prostate cancer, providing new therapeutic targets for patients with bone metastases. Biopsies could guide selection of patients into appropriate therapeutic interventions based on protein levels and RNA expression of desired targets in metastatic disease. Molecular pathology has been an excellent complement to the diagnosis, treatment, and management of primary tumors and could be successfully extended to patients with metastatic lesions.

## Background

Prostate cancers have an improved prognosis in the past two decades, yet metastatic prostate cancer continues to cause high mortality with more than 30,000 deaths in the U. S estimated for 2019 [1]. Most prostate metastases occur in the bone. Treatment for metastatic prostate cancer involves systemic chemotherapy standard of care combined with new and established immunotherapies [2]. Prostate cancer has a unique predilection for metastasis to bone, which most commonly presents as blastic or sclerotic bone lesions, resulting in abnormal growth and stimulation of bone mineralization [3]. However, a smaller subset of lytic or bone-destructive prostate cancers exist and some lesions appear mixed between lytic and blastic phenotypes [4]. These phenotypes in the bone reflect the fundamental tumor and host stroma interaction and can be profoundly changed with the management of the cancer and bone disease as well [5]. Primary prostate tumors are considered “cold”, with low immune cell infiltration and neoantigen expression, making immunotherapy approaches challenging [6]. Metastases form an entirely unique tumor which may result in higher immune cell populations and immune checkpoint activation, reclassifying the metastases as “hot” and more receptive to immunotherapy [7]. There exists mixed opinion on the diagnosis of metastatic tissue, especially in bone, because it can be painful and potentially unnecessary if the diagnosis does not alter treatment [8]. Distinctions in lytic and blastic disease may represent a useful therapeutic approach for managing both the cancer and bone disease of prostate cancer patients.

## Results and discussion

**Fig. 1:**
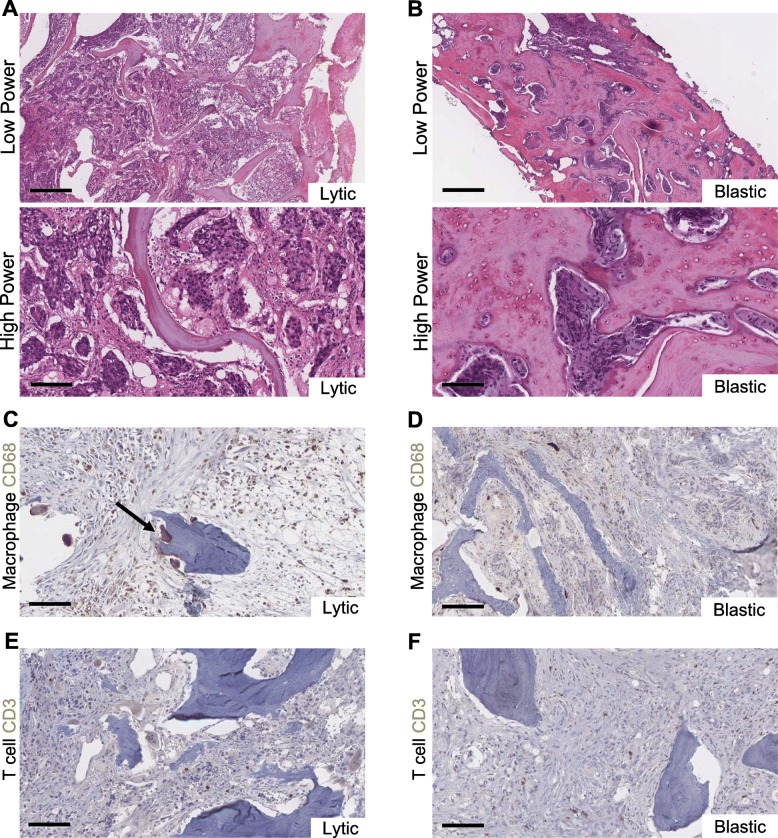
Distinct histopathology of blastic and lytic prostate cancer in bone. **a**, **b** Hematoxylin and Eosin (H&E) staining highlight the appearance of prostate cancer in bone with lytic type metastases that have destructive appearance at bone, while blastic metastases indicate new bone and mineralization with excess matrix and collagen buildup. **c**, **d** CD68 immunohistochemistry (IHC) can identify macrophages as well as other cell types including osteoclasts (black arrow). **e**, **f** T cells are visualized for their location by IHC for CD3 which indicate that diverse sets of T cells exist in both lytic and blastic metastases. Scale Bars = 500 μm for low power and 100um for high power and IHC

We investigated a collection of decalcified formalin-fixed and paraffin-embedded (FFPE) human archived bone tissue samples containing prostate cancer with features of lytic or blastic disease. The deidentified patient cohort underwent varied degrees of treatment with hormone therapy, chemotherapy, radiation, as well as bone disease therapies (Additional file 1: Table S1). Due to limited available patient records, the duration of treatments are unknown. However, the diversity of treatment approaches in the patient samples reflect the challenging variables clinicians encounter when treating aggressive metastatic prostate cancer. The histopathology of these two types of prostate cancers exhibited distinct compositions of tumor and stroma such that the lytic tumor had fundamentally less bone in the tissues, while the blastic lesion contains much more bone and mineralized matrix deposition (Fig. 1a, b, Additional file 1: Figure S1A-J). A hallmark of lytic bone destruction is the increased activation of osteoclast resorption. Osteoclasts stain positively for CD68 because of their myeloid-derived nature that also stains a diverse macrophage population in the bone [9]. Lytic prostate cancers in bone possess CD68-positive osteoclasts and a large collection of macrophage cell types, which are more abundant than in blastic lesions (Fig.1c, d). Primary prostate cancers are considered to be ‘cold’ immunologic tumors and the availability of T cells have been questioned in metastatic disease. We observed that both lytic and blastic prostate cancers in bone have dispersed but sporadic T cell populations as evidenced by immunohistochemistry (IHC) for CD3 (Fig. 1e, f).

**Fig. 2:**
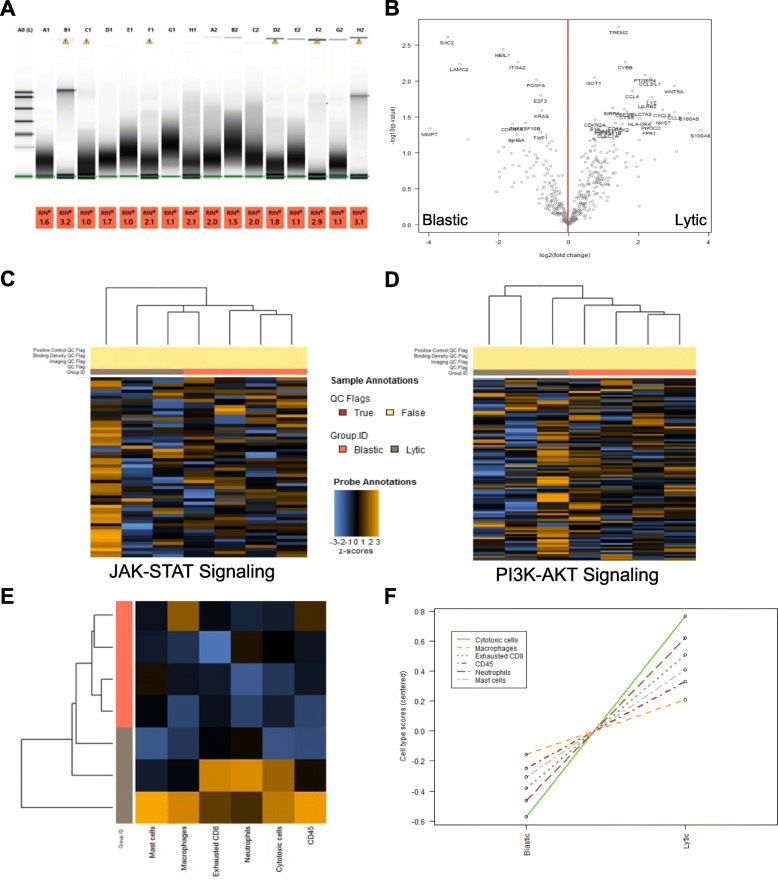
Gene expression from decalcified FFPE prostate cancer in bone. **a** 16 FFPE derived RNA samples (6 lytic and 10 blastic) were analyzed on an Agilent Tape Station for concentration and integrity to produce RNA Integrity Scores (RIN). **b** 3 lytic and 4 blastic samples contained sufficient RNA (25-100 ng) to endure adequate probe coverage of the NanoString Human Immune Oncology 360 gene expression panel. Differential expression revealed a list of significantly upregulated (moving right) and downregulated genes (moving left) in lytic prostate cancer metastases compared to blastic types. **c** Blastic samples were enriched for JAK-STAT pathway genes while (**d**) Lytic samples were enriched for PI3K-AKT gene expression. **e**, **f** Lytic samples based on gene expression demonstrate increased immune cell populations relative to blastic samples. Graphs created using Advanced Analysis module from NanoString nSolver application

A profound difficulty in studying metastatic prostate lesions in bone is not just the limited sample availability, but also the nature of bone, which is decalcified in harsh acid in order to generate suitable tissue sections [10]. Acid decalcification degrades nucleic acids, resulting in poor quality DNA and RNA, making Next Generation Sequencing (NGS) approaches and real-time qPCR difficult if not impossible. We isolated RNA from 20uM-thick sections from demineralized FFPE tissue blocks and found that almost all RNA was of extremely poor quality (Fig. 2a, Additional file 1: Figure S2A-B). Total RNA (25-100 ng) was used with a NanoString Human Immune Oncology 360 gene expression panel, which overcomes the limitations of NGS strategies that require higher quality RNA. Overall, probe coverage was excellent for most samples. Genes with greater than double the counts of the median negative control in 50% or more of the samples were used for gene expression analysis (Additional file 1: Figure S3A-B). Housekeeping genes used for normalization were expressed at moderately high expression levels and exhibited low variance among lytic and blastic samples (Additional file 1: Figure S3C). The frequency of statistical significance was evenly distributed across *p*-values when lytic samples were compared to blastic samples (Additional file 1: Fig. 3d).

**Fig. 3:**
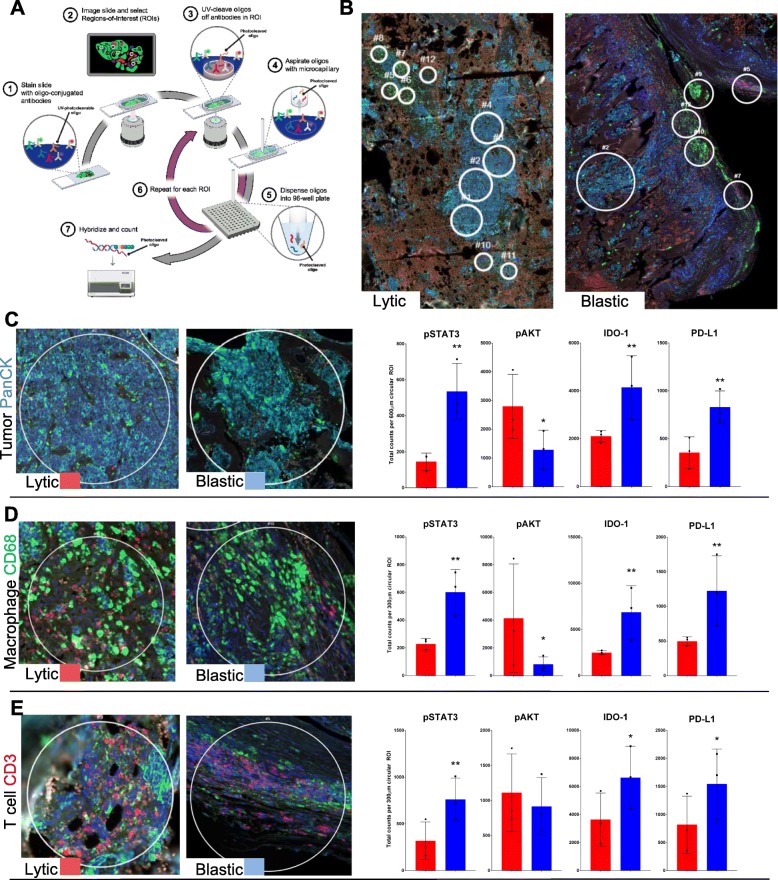
Digital Spatial Profiling of the microenvironment for prostate cancers in bone. **a** Graphic depiction of the process for Digital Spatial Profiling (DSP) whereby tissue sections are selected for Regions of Interest (ROI) and profiled for antibody labeled detection. **b** Fluorescence image of ROI selection of lytic and blastic prostate cancer in bone where tumor (PanCK-cyan) ROIs are 600 μm circles and T cell (CD3-magenta) and Macrophage (CD68-green) ROIs are 300 μm. **c** Tumor ROIs stained cyan with Pan-Cytokeratin (PanCK) were measured for significant antibody presence in 3 patients with at least 4 ROIs per patient. **d** Macrophage ROIs were selected from regions excluding tumor cells and osteoclasts stained with CD68 antibody. **e** T Cell ROIs were selected from regions with high density T cell staining by CD3 antibody. * indicates statistical significance p = < 0.05 and ** indicates p = < 0.001 by Mann-Whitney test. Lytic samples are colored in red and blastic samples are colored in blue for all graphs

A complete list of significant genes enriched for lytic samples compared to blastic samples, sorted by p-value and gene set association, revealed molecular distinctions of lytic and blastic metastatic prostate cancer (Additional file 1: Table S2). Enrichment for Gene Set Analysis (GSA) indicated that lytic type metastases have enriched myeloid compartment genes whether directed or undirected for cancer subtype (Additional file 1: Table S3). Differential expression analysis identified genes such as TREM2, CYBB, PTGER4, WNT5A, and S100A9 were significantly increased in lytic type metastases (Fig. 2b). Genes more commonly associated in blastic samples were SHC2, NEIL1,, ITGA2, LAMC2 and MMP7 (Fig. 2b, Additional file 1: Table S2). Unsupervised clustering of genes associated with distinct signaling pathways revealed enrichments for JAK-STAT signaling in blastic type prostate cancers (Fig. 2c). Alternately, with unsupervised gene clustering, lytic types were enriched for PI3K-AKT signaling (Fig. 2d). The NanoString Human Immune Oncology 360 gene expression panel also suggests which types of cells are enriched in a tissue based on gene expression. Lytic type metastatic prostate cancer had increased cytotoxic cells, macrophages, exhausted CD8 cells, CD45 immune cells, neutrophils, and mast cells (Fig. 2e, f). The increase in immune cells in lytic disease are associated with fewer bone cells by gross histological analysis due to their destruction and replacement by tumor and stromal infiltrates.

It is common to observe increases in distinct immune cells of lytic type prostate cancers in bone. The degradation of bone results in a tissue compartment replacing bone with cells involved in reactive stromal remodeling [11]. To address the distinct tissue heterogeneity, we enrolled in the Technology Access Program (TAP) with NanoString Inc. to perform Digital Spatial Profiling (DSP). DSP was used to investigate distinct components of the tumor and stromal microenvironment. DSP (now commercially available as GeoMx®) allows for spatial analysis of Regions of Interest (ROIs) by staining standard FFPE slides with oligo conjugated antibodies that can be UV cleaved and digitally counted for identified ROIs. These ROIs were manually selected for lytic and blastic prostate cancers guided by immunofluorescence (IF) microscopy (Fig. 3a.). Uniform circular ROIs were selected at 600 μm diameter for tumors, while 300 μm diameter circles were selected for adjacent stroma lacking tumor containing CD68 macrophages or CD3 enriched T cells (Fig. 3b). Three lytic and three blastic patient samples containing tumor within bone were selected such that each case could have four ROIs extracted from tumor, macrophage, and T cell enriched areas. A 33-antibody panel labeled with digital barcodes that could be measured utilizing the NanoString nCounter platform for each ROI isolated was performed (Additional file 1: Table S7). The antibody panel covered cell signaling, immune cell profiling, and immune checkpoint markers to assess differences in blastic and lytic tumor induced bone disease microenvironments. ROIs were selected by IF and antibody barcode staining for pan-cytokeratin (PanCK), CD68, and CD3 which allowed for a digital count to confirm the enrichment for the stain used in ROI selection for all three antibodies.

Tumor ROIs of metastatic prostate cancer in patient bone samples were identified by PanCK staining, and demonstrated reduced cytokeratin expression in lytic type samples not only by IF staining but from digital counts of PanCK antibody (Fig. 3c, Additional file 1: Table S4). Tumor signaling pathway alterations resulted in increased pSTAT3 in blastic samples and increased pAKT in lytic samples. Blastic samples were enriched for multiple checkpoint inhibitor targets compared to lytic samples, including B7-H4 VTCN1, PD-L1, PD-1, VISTA, OX40L, IDO-1 and ICOS CD278. Staining for CD68 macrophage ROIs in metastatic bone demonstrated a significant increase in pSTAT3 in blastic samples (Fig. 3d, Additional file 1: Table S5). Lytic bone disease exhibited a significant increase in pAKT. Checkpoint inhibitor targets B7-H4 VTCN1, PD-L1, PD-1, OX40L, and IDO-1 were high in blastic compared to lytic specimens. Contrary to the tumor ROIs, the immune checkpoint ICOS CD278 was not significantly increased in blastic type samples. T cell enriched CD3 positive ROIs did not have significant difference in pAKT levels but resulted in increased pSTAT3 for blastic type metastases (Fig. 3e, Additional file 1: Table S6). Interestingly, lytic prostate cancers did have increased B7-H3, but in blastic type metastases immune checkpoint markers were not extensively enhanced, with only B7-H4 VTCN1, PD-L1, and OX40L significantly increasing. pSTAT3 signaling was the most universal distinction for blastic and lytic types in all tissue compartments queried and could be seen by standard IHC in both the tumor and stroma (Additional file 1: Figures S4).

## Conclusions

The pathological diagnosis of lytic or blastic disease can be first observed by radiologic reports that detect changes in abnormal bone content. However, because there are no distinct treatment guidelines for metastatic prostate cancer with tumor induced bone diseases, a tissue biopsy is not universally performed [10]. The biopsy diagnosis aids in confirming hormone status and the presence of neuroendocrine features but may not affect the treatment plan for the patient. Currently, National Comprehensive Cancer Network guidelines for M1 castration-resistant prostate cancer with metastases in bone list no therapy guidance for distinguishing between lytic type or blastic type tumor induced bone disease [12]. Tissue biopsies from the bony lesion may allow for precision-based medicine to assess the intrinsic tumor lesion without relying on diagnoses using the primary tumor lesions, which could have been removed decades prior to the metastatic lesion. The distinct pathology of the tissue biopsy may allow for identification of targeted therapy approaches and guide selection for appropriate clinical trials [10].

Prostate tumors and especially metastatic disease are considered on the ‘cold’ spectrum of inflammation [6]. Therapies aimed at reactivating T cells are difficult because sufficient T cell populations of cells are not always available. We show that both lytic and blastic tumors have T cell populations in the bone (Fig. 1e, f). Macrophages are commonly visualized by IHC for CD68, which reflects a diverse class of myeloid derived cells, and are increased in lytic disease (Fig. 1c) [13, 14]. Smaller numbers of distinct macrophages can be seen in blastic metastases with noticeable absence of osteoclasts adjacent to bone, highlighting the lack of demineralization and resorption of bone. The presence of macrophages and T cells in conventionally “cold” prostate cancer bone metastases merit reappraisal of the notion that “hot” and “cold” tumor classification does not solely depend on mutation burden, but is also determined by immune cell infiltration and protein expression. A more comprehensive understanding of the dynamic myeloid-bone interaction during tumor induced bone disease has just begun to emerge as a mechanism of disease progression [15].

The ability to perform genomic analyses on RNA and DNA from bone have largely been achieved by establishing careful protocols to avoid over fixation, but most importantly avoiding decalcification of bone in strong acids [10]. Only recently, even with avoiding harsh acids, have NGS strategies shown promise. The ability to use FFPE bone tissue that has been decalcified and contains degraded RNA/DNA opens up a broader range of samples that can be accessed from decades prior, resulting in increased access to usable patient samples and detailed longitudinal follow up for patient outcomes. The advancement of biopsies with specialized collection protocols that facilitate molecular analyses can help guide the ever-growing list of new therapeutic strategies [16]. These new findings suggest that empirical archived data is now worthwhile for investigating molecular pathology. Using gene expression from a biopsy to inform the clinical partner from a molecular diagnostic test could potentially serve as a selection tool to target a given population of T cells or myeloid suppressive cells [17]. Advances in gene expression profiling have already led to predicting outcomes of immune oncology for PD-1/PD-L1 blockade treatments, which may be useful in distinct prostate cancer patients [18].

Many prostate cancers are of a blastic or sclerotic type, resulting in large portions of biopsies filled with bone mineral and matrix [19]. This study utilized the emerging DSP technology to address the ROIs of specific tumor and stroma, so that a fundamental molecular nature of these populations can be assessed [20]. Recent oncology studies using DSP have shown that expression of checkpoint targets such as PD-L1 can be monitored in high-risk melanoma patients for response to effective immunotherapy regimens [21, 22]. These studies highlight the new capacity to evaluate the immune microenvironment within or juxtaposed to the tumor. The ability to isolate proteomic and genomic data from defined tissue areas in a given pathology without the need to destroy or cut the tissue is valuable, allowing for follow up investigations. Prostate cancer patient lytic and blastic bone metastases have previously been pathologically discernable, but with this study can now be identified with molecular and cellular distinctions. For each individual patient profiled, a rank category of most promising targets of signaling molecules such as pAKT or pSTAT3 of which targeted therapies are currently available could be rationally employed [23, 24]. Checkpoint inhibitors continue to expand and combinations of multiple inhibitors increasingly improve outcomes [25]. In metastatic disease, the ability to understand which drug combinations, based on genomic and proteomic enrichments, may have a profound ability to sort patients with the best treatments [26].

## Materials and methods

### Histology and immunohistochemistry (IHC)

Deidentified surgical specimens from bone containing prostate cancer that underwent fixation and decalcification restricted to bone without soft tissue involvement were processed. Tissue blocks were sectioned at 4 μm for 10 slides. Then 5 sections of 20 μm were cut to produce ‘scrolls’ of rolled up paraffin containing the tissue to be placed immediately in RNase/DNase free tubes for further nucleic acid isolation. An additional 10 slides at 4 μm were cut, with the first and last slide stained for Hematoxylin and Eosin (H&E) to compare changes in morphology. Unstained slides were baked for one hour at 60 degrees prior to xylene paraffin removal and rehydration of tissue in ethanol. Antigen retrieval was performed in Citrate pH 6.0 in a pressure cooker (NxGen, BioCare Medical). Primary antibodies (CD3, CD68 from DAKO and pSTAT3 TYR705 from Cell Signaling) were detected with HRP conjugated polymer and developed with DAB chromogen (Vector Labs). Slides were counterstained with Hematoxylin QS (Vector Labs). All bright field IHC and H&E were scanned at 40X (0.22 μm/pixel) magnification using a ScanScope XT System (Aperio Technologies).

### RNA isolation and gene expression

16 FFPE derived bone tissue containing prostate cancer (6 lytic and 10 blastic) were sectioned to 20 μm and 3–5 scrolls were placed in RNase free tubes where RNA was isolated using Qiagen FFPE All-prep RNA/DNA Extraction kit. RNA was analyzed on an Agilent Bioanalyzer for concentration and degradation to produce RNA Integrity Scores (RIN). Gene expression was performed using the NanoString Human Immune Oncology 360 gene expression panel XT v1.0. 25-100 ng of RNA was used per sample and run on the nCounter Sprint Profiler following manufacturers recommendations (NanoString Inc.). nSolver™ analysis software v4.0 was used for RCC file analysis. Advanced Analysis (AA) modules were used for differential expression, pathway enrichments in lytic vs blastic cases as well as cell profiling and Gene Set Analysis (GSA).

### Digital spatial profiling

Multiplex IHC was performed using Digital Spatial Profiling (DSP) with a nuclear stain, and antibodies to identify tumor (Pan-Cytokeratin), T cells (CD3) and macrophages (CD68). Slides were sent to NanoString (Seattle WA, USA) as part of their Technology Access Program (TAP) where slide staining was optimized on the DSP system (schematic overview Fig. 3a). Regions of Interest (ROI) were selected such that tumor (PanCK) was adjacent to bone for tumor ROI. Macrophage (CD68) and T cell (CD3) ROIs were selected away from tumor at least one 20x field of view (FOV). ROIs for tumor were circular at 600 μm diameter and T cell and Macrophage ROIs were circular 300 μm diameters. ROIs were illuminated with ultraviolet light to release the barcoded oligos corresponding to their 33 ascribed antigen targets (Additional file 1: Table S7). After all ROIs were processed and barcoded oligos collected, digital counting was performed with the nCounter and processing of counts using the DSP App v5.3. Raw counts from barcoded oligo probes derived from protein probes were normalized with internal spike-in controls to account for system variation. Normalized digital counts are displayed in Additional file 1: Tables S4, S5 and S6.

### Statistical analyses

Analyses were performed using GraphPad Prism (version 7.04 for Windows; GraphPad Software Inc.). All statistical tests used a cutoff *P*-value of 0.05 for significance and were two-sided. Student’s t-test was performed for ROIs digital counts.

## Supplementary information

**Additional file 1: Figure S1.** Additional histology images. (A-E) Representative low power (4X) magnification of H&E stained patient samples from bone tissue containing lytic type prostate cancer metastases. (F-J) Representative low power (4X) magnification of H&E stained patient samples from bone tissue containing blastic type prostate cancer metastases. Scale Bars = 500 μm. **Figure S2.** RNA quality derived from decalcified FFPE tissues. (A) Detailed quantification of RNA isolated from 20 μm scrolls collected for RNA extraction and quantified with 1ul using a NanoDrop instrument. (B) Agilent Tape station results from 16 FFPE derived patient samples. (C) Histogram view of electronic ladder for RNA integrity assessment indicating quality for intact RNA species. (D) Representative histogram of degraded RNA used in this study with still intact smaller species of RNA suitable for probe binding for gene expression. **Figure S3.** NanoString nSolver Heatmaps of raw and total data behavior. (A) Heatmap of the raw counts. Overview of how probe counts range in raw expression levels across samples. Samples that lack high level of probe expression (e.g. counts > 100) may indicate failure. Probes are called detected if they have more than double the counts of the median negative control. (B) Heatmap of the normalized data, scaled to give all genes equal variance, generated via unsupervised clustering. Orange indicates high expression; blue indicates low expression (C) Variance vs. Mean normalized signal plot across all targets/probes. Each gene’s variance in the log-scale, normalized data is plotted against its mean value across all samples. Highly variable genes are indicated by gene name. Housekeeping genes are color coded according to their use in normalization. (D) For each covariate included in the analysis, a histogram of p-values testing each gene’s univariate association with the chosen covariate is displayed. Covariates with largely flat histograms have minimal association with gene expression; covariates with histograms with significantly more mass on the left are either associated with the expression of many genes or are confounded with a covariate that is associated with the expression. Low p-values indicate strong evidence for an association. **Figure S4.** IHC additional sample for pSTAT3 Y705. (A-B) pSTAT3 (Y705) identifies active JAK-STAT signaling in the nucleus of tumor and stroma. Scale Bars = 500 μm for low power and 100 μm for high power and IHC. **Table S1.** Patient Sample Treatment History and Characteristics. **Table S2.** Differential Expression of Lytic vs. Blastic RNA. Table presenting the most statistically significant differentially expressed genes with the lytic samples as covariate. For categorical covariates, a gene is estimated to have 2^(log fold change) times its expression in baseline samples, holding all other variables in the analysis constant. **Table S3.** Gene Set Analysis (GSA) Undirected and directed global significance scores table. The results of differential expression testing are summarized at the gene set level. Each gene set’s most differentially expressed genes are identified, and the extent of differential expression in each gene set is summarized using a global significance score. Each sample’s global significance scores and directed global significance. The global significance score is calculated as the square root of the mean squared t-statistic for the genes in a gene set, with t-statistics coming from the linear regression underlying the differential expression analysis. **Table S4.** DSP Normalized antibody counts: Tumor ROI. Antigen table refers to the specific antibody used in Pan-Cytokeratin guided staining of 600 μm circular Regions Of Interest (ROI) of tumor. Averages across blastic and lytic samples for each antigen, with standard deviation and statistical significance indicated by Students t-test. **Table S5.** DSP Normalized antibody counts: Macrophage ROI. Antigen table refers to specific antibody used in CD68-guided staining of 300 μm circular Regions Of Interest (ROI) of macrophages. Averages across blastic and lytic samples for each antigen with standard deviation and statistical significance indicated by Students t-test. **Table S6.** DSP Normalized antibody counts: T Cell ROI. Antigen table refers to specific antibody used in CD3-guided staining of 300 μm circular Regions Of Interest (ROI) of T cells. Averages across blastic and lytic samples for each antigen with standard deviation and statistical significance indicated by Students t-test. **Table S7.** Antibody panel for Digital Spatial Profiling.

## Data Availability

All data are available in the paper or supplement. Materials are partially restricted by sample availability and lot consistency of reagents. The RNA and probe datasets are available from the corresponding author by request for use with nSolver software provided free from Nanostring Inc. The DSP data that support the findings of this study are available from NanoString and the corresponding author.

## Abbreviations

AA: Advanced Analysis
DSP: Digital Spatial Profiling
FFPE: Formalin-Fixed Paraffin-Embedded
FOV: Field of View
GSA: Gene Set Analysis
H&E: Hematoxylin and Eosin
IF: Immunofluorescence
IHC: Immunohistochemistry
NGS: Next Generation Sequencing
PanCK: Pan-cytokeratin
RIN: RNA Integrity Score
ROI: Regions of Interest
TAP: Technology Access Program
