# Supplementary material for: Distinct tumor microenvironments of lytic and blastic bone metastases in prostate cancer patients
Source: J Immunother Cancer. 2019 Nov 8;7:293. doi: 10.1186/s40425-019-0753-3 (PMC6839115; doi:10.1186/s40425-019-0753-3)
Supplement: Supplementary file 1 — Additional file 1: Figure S1. Additional histology images. (A-E) Representative low power (4X) magnification of H&E stained patient samples from bone tissue containing lytic type prostate cancer metastases. (F-J) Representative low power (4X) magnification of H&E stained patient samples from bone tissue containing blastic type prostate cancer metastases. Scale Bars = 500 μm. Figure S2. RNA quality derived from decalcified FFPE tissues. (A) Detailed quantification of RNA isolated from 20 μm scrolls collected for RNA extraction and quantified with 1ul using a NanoDrop instrument. (B) Agilent Tape station results from 16 FFPE derived patient samples. (C) Histogram view of electronic ladder for RNA integrity assessment indicating quality for intact RNA species. (D) Representative histogram of degraded RNA used in this study with still intact smaller species of RNA suitable for probe binding for gene expression. Figure S3. NanoString nSolver Heatmaps of raw and total data behavior. (A) Heatmap of the raw counts. Overview of how probe counts range in raw expression levels across samples. Samples that lack high level of probe expression (e.g. counts > 100) may indicate failure. Probes are called detected if they have more than double the counts of the median negative control. (B) Heatmap of the normalized data, scaled to give all genes equal variance, generated via unsupervised clustering. Orange indicates high expression; blue indicates low expression (C) Variance vs. Mean normalized signal plot across all targets/probes. Each gene’s variance in the log-scale, normalized data is plotted against its mean value across all samples. Highly variable genes are indicated by gene name. Housekeeping genes are color coded according to their use in normalization. (D) For each covariate included in the analysis, a histogram of p-values testing each gene’s univariate association with the chosen covariate is displayed. Covariates with largely flat histograms have minimal association wi [file 40425_2019_753_MOESM1_ESM.pdf]

Supplemental Data Ihle

The following is supplemental data to be submitted with primary data:

Supplemental Figure 1 Ihle

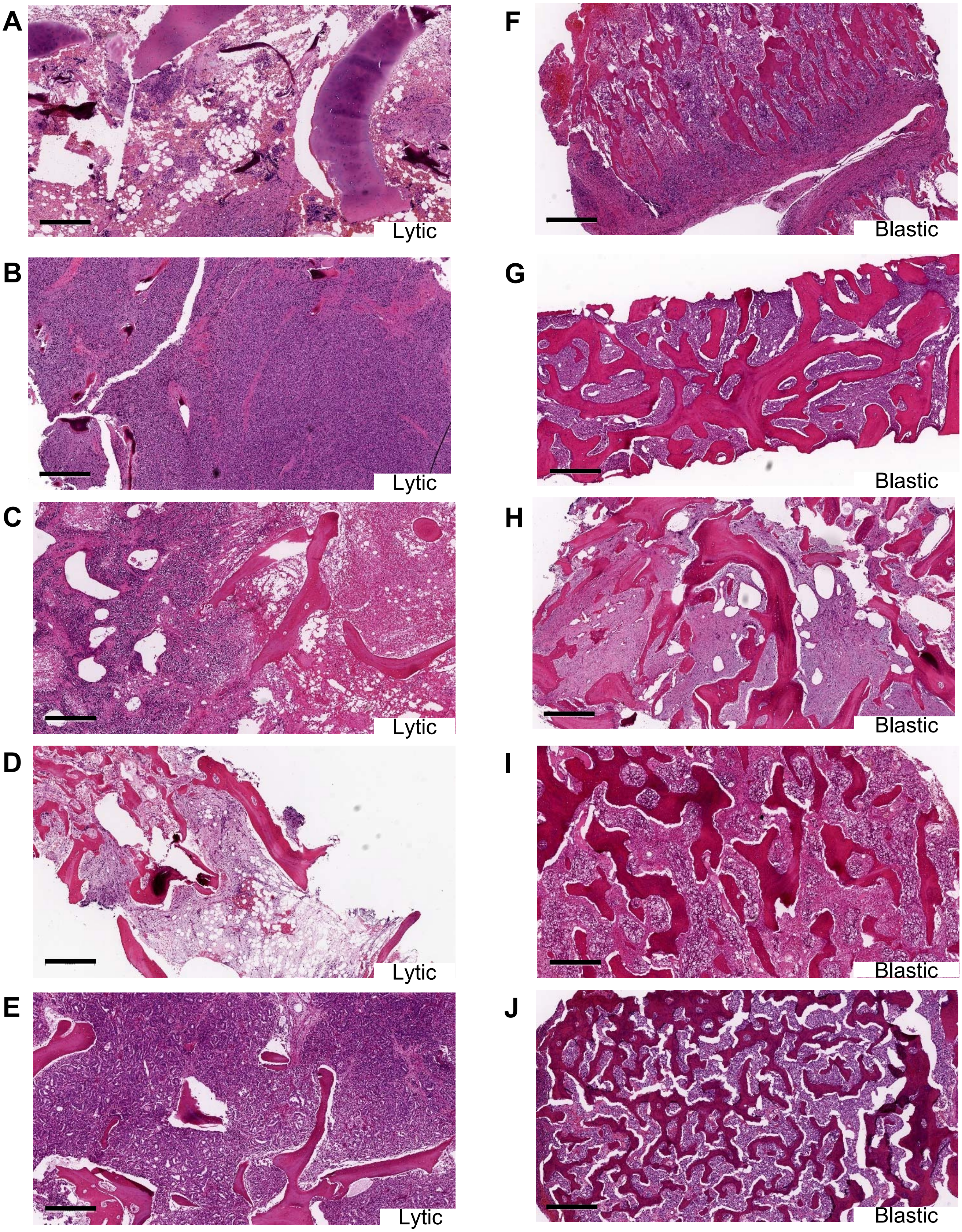

Supplemental Figure 2 Ihle

A

Sample ID	Nucleic Acid Conc.	Unit	A260	A280	260/280	260/230	Sample Type	Factor
RNA 06	66.6	ng/µl	1.664	0.91	1.83	0.17	RNA	40
RNA 13	10.6	ng/µl	0.266	0.169	1.57	0.04	RNA	40
RNA 21	52.1	ng/µl	1.303	0.779	1.67	0.24	RNA	40
RNA 27	155	ng/µl	3.875	2.009	1.93	0.31	RNA	40
RNA 32	378.8	ng/µl	9.47	4.582	2.07	0.55	RNA	40
RNA 33	30.4	ng/µl	0.759	0.35	2.17	0.05	RNA	40
RNA 33A	401.9	ng/µl	10.048	4.941	2.03	0.65	RNA	40
RNA 35	15.2	ng/µl	0.381	0.185	2.06	0.04	RNA	40
RNA 36	44	ng/µl	1.1	0.654	1.68	0.07	RNA	40
RNA 47	10	ng/µl	0.25	0.147	1.7	0.05	RNA	40
RNA 48	17.8	ng/µl	0.445	0.247	1.8	0.05	RNA	40
RNA 50	191.1	ng/µl	4.778	2.415	1.98	0.33	RNA	40
RNA 51	55.3	ng/µl	1.383	0.689	2.01	0.12	RNA	40
RNA 52	130.9	ng/µl	3.274	1.664	1.97	0.26	RNA	40
RNA 55	6.6	ng/µl	0.166	0.1	1.66	0.06	RNA	40
RNA 56	35	ng/µl	0.875	0.496	1.76	0.1	RNA	40

B

Sample Info

Well	RINe	28S/18S (Area)	Conc. [pg/µl]	Sample Description	Alert	Observations
A0	-	-	3850	Electronic Ladder		Ladder
A1	1.6	-	1500	06		
B1	3.2	-	328	13	⚠	RNA concentration outside recommended range for RINe
C1	1.0	-	671	21	⚠	RNA concentration outside recommended range for RINe
D1	1.7	-	5290	27		
E1	1.0	-	20400	32		
F1	2.1	-	858	33	⚠	RNA concentration outside recommended range for RINe
G1	1.1	-	18800	33A		
H1	2.1	-	1090	36		
A2	2.0	-	5540	50		
B2	1.5	-	9680	51		
C2	2.0	-	6660	52		
D2	1.8	-	684	56	⚠	RNA concentration outside recommended range for RINe
E2	1.1	-	3400	35		
F2	2.9	-	166	47	⚠	RNA concentration outside recommended range for RINe
G2	1.1	-	1390	48		
H2	3.1	-	339	55	⚠	RNA concentration outside recommended range for RINe

C

A0: Electronic Ladder

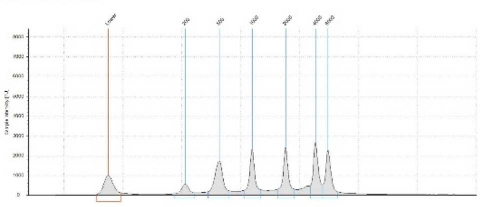

Sample Table

Well	RINe	28S/18S (Area)	Conc. [pg/µl]	Sample Description	Alert	Observations
A0	-	-	3850	Electronic Ladder		Ladder

Peak Table

Size [nt]	Calibrated Conc. [pg/µl]	Assigned Conc. [pg/µl]	Peak Molarity [pmol/l]	%Integrated Area	Peak Comment	Observations
25	700	700	82400	-		Lower Marker
200	195	-	2870	6.33		
500	625	-	3680	20.25		
1000	544	-	1600	17.62		
2000	567	-	833	18.36		
4000	614	-	452	19.91		
6000	541	-	265	17.52		

D

A1: 06

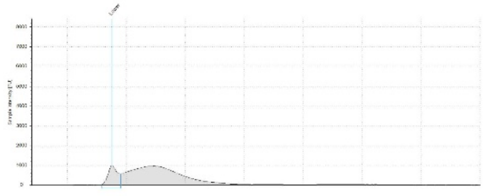

Sample Table

Well	RINe	28S/18S (Area)	Conc. [pg/µl]	Sample Description	Alert	Observations
A1	1.6	-	1500	06		

Peak Table

Size [nt]	Calibrated Conc. [pg/µl]	Assigned Conc. [pg/µl]	Peak Molarity [pmol/l]	%Integrated Area	Peak Comment	Observations
25	700	700	82400	-		Lower Marker

Supplemental Figure 3 Ihle

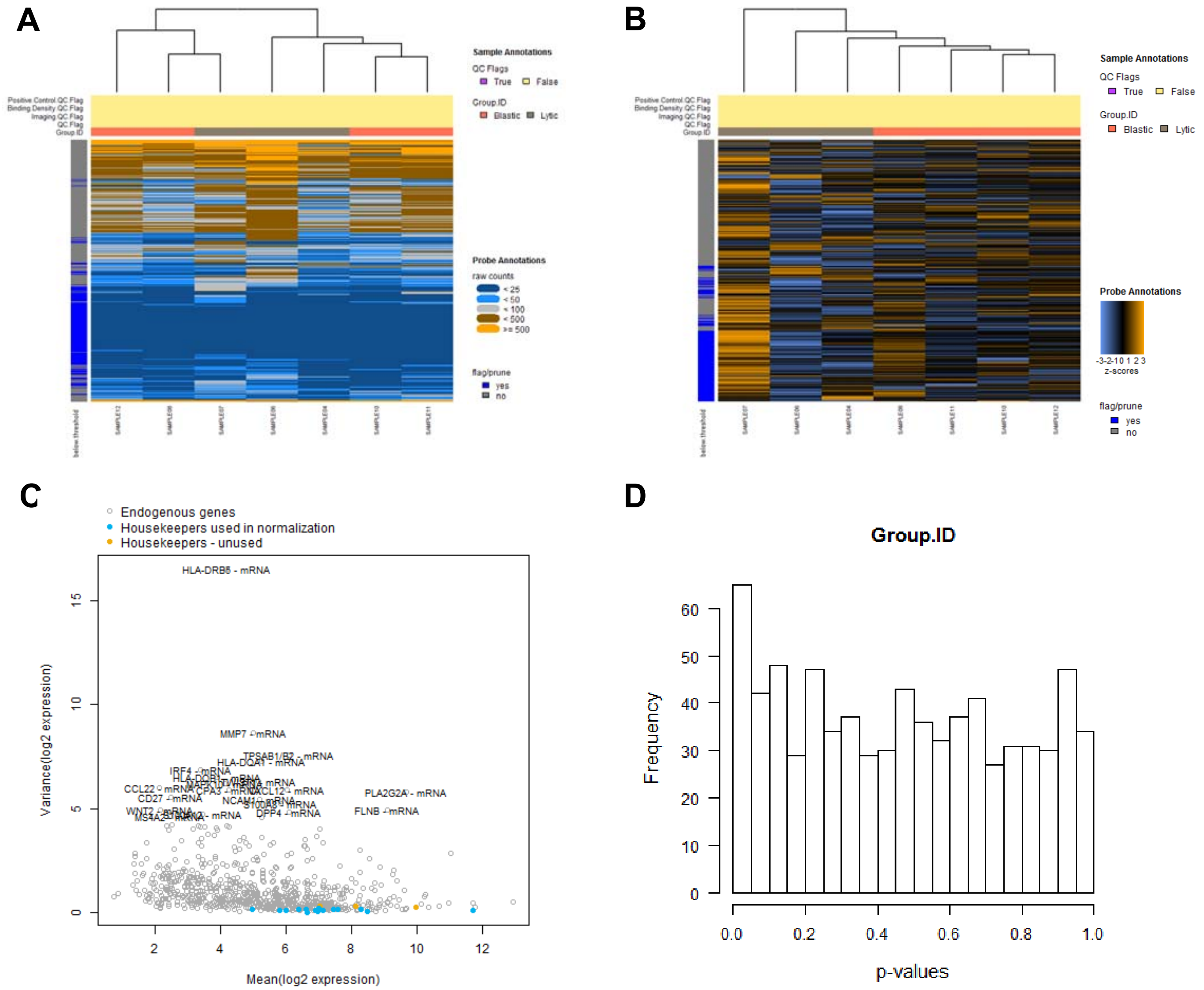

Supplemental Figure 4 Ihle

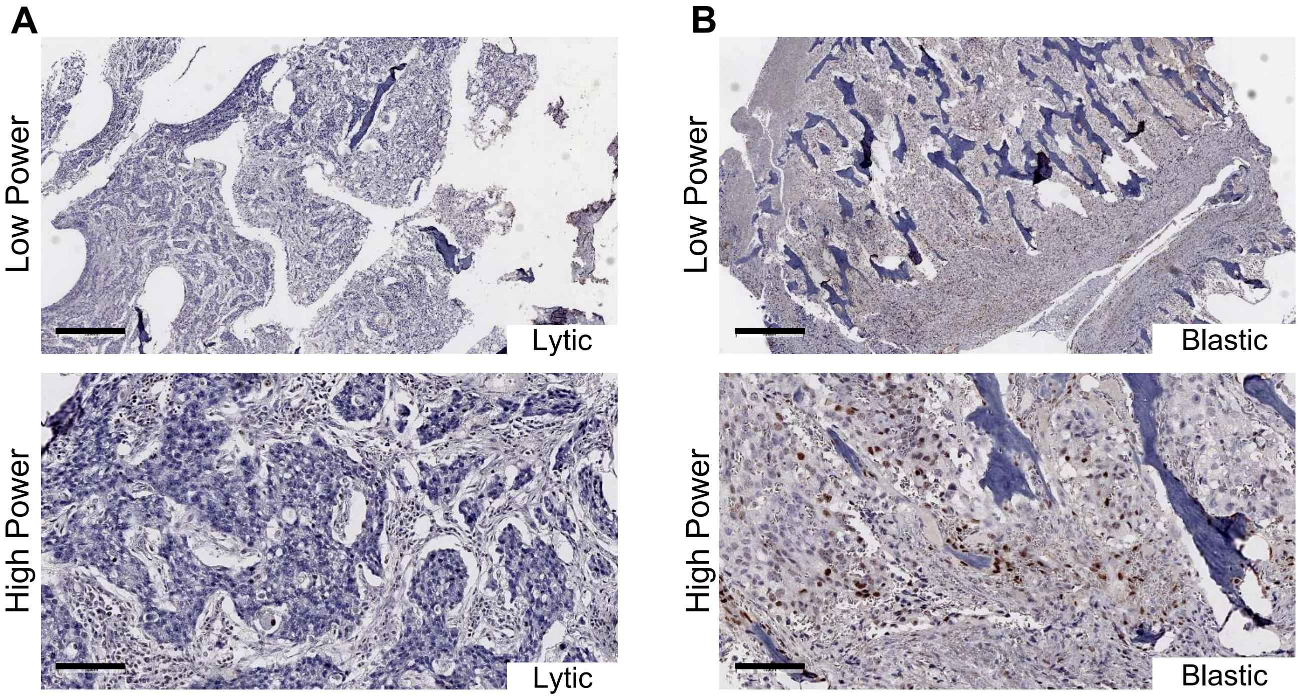

Supplemental Table 1. Patient Sample Treatment History and Characteristics

Metastasis Site		
	Rib	1/15
	Spine	3/15
	Pelvis	2/15
	Femur	5/15
	Humerus	2/15
	Thoracic	1/15
	Orbit	1/15
	Other Bone	2/15
Bone Disease		
	Lytic	5/15
	Blastic	10/15
Hormone Therapy		
	Androgen Deprivation	6/15
	Leuprolide	11/15
	Bicalutamide	3/15
	Degarelix	2/15
	Goserelin	1/15
	Enzalutamide	4/15
	Diethylstilbestrol	1/15
	Abiraterone	3/15
	Megestrol Acetate	1/15
Radiation		
		13/15
Chemotherapy		
	Docetaxel	5/15
	Cabaziletaxel	1/15
	Carboplatin	4/15
	Mitoxantrone	1/15
Osteoporosis		
	Denosumab	5/15
	Ibandronic Acid	1/15
Other		
	Finasteride	1/15
	Unknown patient history	3/15

Supplemental Table 2 Ihle

Gene Name	Log2 fold change	std error (log2)	Linear fold change	P-value	Gene.sets	probe.ID
TREM2-mRNA	1.43	0.236	2.69	0.00176	Myeloid Compartment	NM_018965.3:848
SHC2-mRNA	-3.46	0.613	0.091	0.00243	Cytokine and Chemokine Signaling, MAPK	NM_012435.2:681
NEIL1-mRNA	-1.87	0.364	0.274	0.00365	DNA Damage Repair	NM_024608.2:1675
CYBB-mRNA	1.63	0.348	3.1	0.0054	Antigen Presentation, Hypoxia, Immune Cell Adhesion and Migration, Myeloid Compartment	NM_000397.3:1034
ITGA2-mRNA	-1.45	0.31	0.365	0.0054	Immune Cell Adhesion and Migration, Matrix Remodeling and Metastasis, PI3K-Akt	NM_002203.3:903
LAMC2-mRNA	-3.11	0.674	0.116	0.00574	Matrix Remodeling and Metastasis, PI3K-Akt	NM_005562.2:1027
PTGER4-mRNA	2.19	0.516	4.55	0.00819		NM_000958.2:975
GOT1-mRNA	0.734	0.177	1.66	0.00893	Metabolic Stress	NM_002079.2:568
CCL3/L1-mRNA	2.33	0.566	5.03	0.00919	Cytokine and Chemokine Signaling	NM_021006.5:411
PDGFA-mRNA	-0.912	0.224	0.531	0.0096	MAPK, Matrix Remodeling and Metastasis, PI3K-Akt	NM_033023.4:2390
WNT5A-mRNA	3.02	0.778	8.1	0.0117	Hedgehog Signaling, Wnt Signaling	NM_003392.3:987
CCL4-mRNA	1.82	0.49	3.54	0.0137	Antigen Presentation, Cytokine and Chemokine Signaling, Myeloid Compartment	NM_002984.2:35
E2F3-mRNA	-0.798	0.222	0.575	0.0157	Angiogenesis, Cell Proliferation, Metabolic Stress, Notch Signaling	NM_001243076.2:2707
LYZ-mRNA	2.38	0.673	5.21	0.0166	Myeloid Compartment	NM_000239.2:381
LILRB2-mRNA	2.26	0.657	4.78	0.0186	Costimulatory Signaling, Myeloid Compartment	NM_001278405.1:256
SIRPA-mRNA	1.25	0.386	2.37	0.0234	Cytotoxicity, Myeloid Compartment	NM_001040023.1:3355
SLC7A5-mRNA	2.1	0.657	4.28	0.0242	Metabolic Stress	NM_003486.6:2011
CSF3R-mRNA	1.6	0.502	3.02	0.0247	Cytokine and Chemokine Signaling, JAK-STAT Signaling, Myeloid Compartment, PI3K-Akt	NM_000760.3:2066
CXCL8-mRNA	2.68	0.847	6.41	0.025	Cytokine and Chemokine Signaling, Metabolic Stress	NM_000584.3:170
KRAS-mRNA	-0.766	0.243	0.588	0.0255	Autophagy, Cytokine and Chemokine Signaling, MAPK, Metabolic Stress, PI3K-Akt	NM_004985.3:1790
CTSS-mRNA	1.67	0.539	3.19	0.0267	Antigen Presentation, Matrix Remodeling and Metastasis	NM_004079.3:685
CCL5-mRNA	3.04	0.984	8.22	0.0272	Cytokine and Chemokine Signaling, Myeloid Compartment	NM_002985.2:277
S100A9-mRNA	3.44	1.12	10.9	0.028	Myeloid Compartment	NM_002965.3:137
NKG7-mRNA	2.71	0.92	6.56	0.0319		NM_005601.3:633
HLA-DRA-mRNA	2.03	0.697	4.09	0.0331	Antigen Presentation, Costimulatory Signaling, Immune Cell Adhesion and Migration, Interferon Signaling	NM_019111.3:335
CDKN2A-mRNA	0.763	0.263	1.7	0.0339	Cell Proliferation, Metabolic Stress	NM_000077.4:1052
PIK3CD-mRNA	2.37	0.845	5.19	0.0375	Autophagy, Cytokine and Chemokine Signaling, Hypoxia, Immune Cell Adhesion and Migration, JAK-STAT Signaling, MAPK, Metabolic Stress, PI3K-Akt	NM_001350234.1:664
TNFRSF10B-mRNA	-1.24	0.444	0.423	0.0382	Apoptosis	NM_003842.3:565
CD14-mRNA	1.33	0.477	2.52	0.0382	Apoptosis, MAPK, Myeloid Compartment	NM_001174105.1:1020
SYK-mRNA	0.776	0.279	1.71	0.0388	PI3K-Akt	NM_003177.5:808
CDKN1A-mRNA	-1.6	0.579	0.329	0.0394	Cell Proliferation, Hypoxia, JAK-STAT Signaling, Metabolic Stress, Myeloid Compartment, PI3K-Akt	NM_000389.2:1975
PCK2-mRNA	1.54	0.56	2.92	0.04	Metabolic Stress, PI3K-Akt	NM_001018073.2:1307
DNMT1-mRNA	1.04	0.381	2.05	0.0417	Epigenetic Regulation	NM_001130823.2:2500
TNFRSF1B-mRNA	1.13	0.424	2.19	0.044	NF-kappaB Signaling	NM_001066.2:596
FPR1-mRNA	2.3	0.862	4.93	0.0444	Myeloid Compartment	NM_002029.3:625
MMP7-mRNA	-3.97	1.5	0.0639	0.0459	Matrix Remodeling and Metastasis, Wnt Signaling	NM_002423.3:265
S100A8-mRNA	3.77	1.45	13.6	0.048	Myeloid Compartment	NM_002964.4:365
CLEC7A-mRNA	1.12	0.43	2.17	0.0488	Immune Cell Adhesion and Migration, Myeloid Compartment	NM_197954.2:55

Supplemental Table 3. GSA Global Significance Scores of Differential Expression in Lytic vs. Baseline of Blastic

	Undirected Group	Directed Group
Myeloid Compartment	2.287	2.155
Antigen Presentation	1.624	1.417
Epigenetic Regulation	1.339	1.264
Cytokine and Chemokine Signaling	1.955	1.077
Hypoxia	1.58	1
Immune Cell Adhesion and Migration	1.697	0.958
Costimulatory Signaling	1.429	0.892
Metabolic Stress	1.585	0.891
Hedgehog Signaling	1.613	0.881
Cell Proliferation	1.423	0.796
Interferon Signaling	1.037	0.776
Cytotoxicity	1.079	0.754
JAK-STAT Signaling	1.39	0.693
DNA Damage Repair	1.609	0.485
Autophagy	1.546	0.442
Apoptosis	1.258	0.412
Lymphoid Compartment	1.183	-0.516
Angiogenesis	1.237	-0.905
MAPK	1.511	-0.974
Matrix Remodeling and Metastasis	1.759	-1.07

Supplemental Table 4 Ihle

Supplemental Table 4. DSP Normalized Antibody Counts: Tumor ROI.

Antigen	Lytic														Blastic														p-Value
	Patient 1				Patient 2				Patient 3				Average	ST DEV	Patient 1				Patient 2				Patient 3				Average	ST DEV	
**Pan-Cytokeratin**	11380	11199	13124	10798	2128	2114	2636	2723	1072	332	953	3444	**5158**	**4879**	4324	3759	5369	5880	7719	6862	6759	8352	12631	12501	16588	14934	**8806**	**4282**	**0.0644474**
**CD3**	45	70	181	192	97	110	36	224	154	488	155	240	**166**	**121**	123	135	175	146	267	219	378	374	150	182	188	268	**217**	**88**	**0.2495942**
**CD68**	5257	7635	3888	5520	8434	4335	4733	8038	7964	6293	16503	5626	**7019**	**3362**	1913	1750	2402	1292	10456	9808	15388	19530	4653	8903	10628	15023	**8479**	**6141**	**0.4776333**
**P-AKT (Ser473)**	3824	4017	5055	3372	1066	3486	4080	695	528	299	627	6485	**2794**	**2072**	1036	1448	1371	1395	579	540	605	650	1268	1334	2292	2899	**1285**	**717**	**0.0260781**
**P-STAT3 (Tyr705)**	84	108	86	66	280	99	130	179	136	161	143	258	**144**	**67**	681	969	442	768	432	390	453	433	433	448	483	489	**535**	**177**	**0.0000004**
**Ki-67**	2205	1821	2040	1375	21387	11251	10561	14149	4864	7242	3174	1820	**6824**	**6329**	1539	1409	2118	2395	17199	21846	12658	10372	1411	1839	1466	2575	**6402**	**7247**	**0.8806700**
**Bcl-2**	238	295	223	257	2062	1013	1171	1493	203	457	246	273	**661**	**624**	717	743	1005	838	1841	1803	1899	1787	812	999	739	799	**1165**	**502**	**0.0400674**
**CD45**	239	274	445	741	148	71	50	133	331	359	349	312	**288**	**189**	154	149	213	166	335	249	430	417	304	334	434	657	**320**	**149**	**0.6448369**
**CD4**	848	1147	786	959	1295	772	653	1216	1249	1202	1665	713	**1042**	**302**	359	538	586	370	1553	1332	1887	2162	741	1141	1302	2254	**1186**	**680**	**0.5105971**
**FoxP3**	29	24	27	24	91	33	39	52	45	24	27	188	**50**	**47**	70	91	107	76	123	102	109	110	96	121	102	99	**100**	**16**	**0.0020780**
**GZMB**	582	749	567	574	1252	986	797	1350	737	747	678	548	**797**	**267**	1013	1198	1546	1211	3531	3072	2827	2587	766	860	965	921	**1708**	**1000**	**0.0058989**
**CD44**	305	478	355	501	1960	1164	1085	3134	924	937	964	183	**999**	**832**	564	1175	616	885	686	506	910	1096	859	1016	885	2138	**945**	**430**	**0.8418154**
**CD8A**	88	70	301	557	229	182	101	356	142	397	137	215	**231**	**147**	133	150	245	159	419	343	523	623	123	155	191	140	**267**	**170**	**0.5889857**
**CD45RO**	253	321	245	299	208	213	132	172	285	361	291	218	**250**	**65**	187	200	226	223	318	338	368	412	288	334	311	332	**295**	**71**	**0.1220617**
**CD11c**	2339	3215	1534	2103	1869	1367	1404	2955	5999	4972	12893	1812	**3538**	**3284**	1043	854	1224	797	2565	2619	3395	4477	2024	4175	4170	5633	**2748**	**1623**	**0.4626627**
**CD14**	276	274	251	266	160	94	81	148	126	149	149	255	**186**	**73**	155	217	302	164	322	261	340	312	176	324	322	353	**271**	**73**	**0.0093053**
**CD163**	137	159	173	201	166	137	141	167	221	205	262	228	**183**	**40**	56	82	102	44	292	262	431	435	54	101	117	179	**180**	**142**	**0.9357910**
**CD66B**	736	81	201	254	54	28	35	50	258	98	63	262	**177**	**199**	51	70	85	60	94	64	171	79	66	72	163	175	**96**	**46**	**0.1844174**
**CD56**	3219	1935	848	1410	347	117	102	527	995	2098	4106	341	**1337**	**1284**	5533	14043	9289	6310	448	319	348	483	10036	20064	17002	26181	**9171**	**8650**	**0.0051829**
**HLA-DR**	1411	1345	1181	1117	605	981	503	1374	3838	1849	2702	1175	**1507**	**929**	592	857	1333	767	2401	1221	1774	2532	1551	2223	2959	6577	**2065**	**1608**	**0.3085843**
**B7-H3**	67229	88204	57082	60250	35405	25110	28186	41767	109585	63428	99911	58194	**61196**	**27090**	63157	91750	121730	73357	60132	57971	45690	50094	24472	38684	64778	84512	**64694**	**25919**	**0.7495997**
**B7-H4 VTCN1**	46	77	50	54	169	62	80	106	92	40	69	303	**96**	**74**	165	237	294	209	277	239	244	264	171	249	229	205	**232**	**39**	**0.0000115**
**PD-L1**	171	206	179	181	542	262	280	402	307	203	243	1291	**355**	**314**	595	771	1057	684	984	969	1069	1056	569	779	724	704	**830**	**186**	**0.0001754**
**PD1**	37	47	32	32	148	70	56	94	57	42	43	282	**78**	**72**	95	116	161	109	166	144	147	153	170	230	257	212	**163**	**49**	**0.0027366**
**STING TMEM173**	2377	1554	2517	2708	10958	6905	7406	6737	1459	1444	1572	2976	**4051**	**3138**	1551	1744	2021	2180	7780	6086	8688	9981	1525	2437	3373	3425	**4233**	**3065**	**0.8873863**
**VISTA**	481	346	436	403	1020	499	525	635	491	423	546	347	**513**	**180**	261	305	367	209	708	460	606	616	4140	7464	8499	17136	**3398**	**5240**	**0.0697749**
**OX40L**	24	22	22	19	130	57	57	78	58	25	42	317	**71**	**84**	123	147	190	132	236	207	217	241	117	190	168	136	**175**	**44**	**0.0009499**
**Beta-2-Microglobulin**	28009	30940	27780	27605	5022	2908	2068	5152	7596	5047	9129	6910	**13181**	**11558**	7240	8391	8273	6259	10775	11421	13398	14767	11747	16648	22354	33349	**13719**	**7647**	**0.8942560**
**IDO-1**	1645	1864	2047	1746	2849	1701	1853	2797	1777	1471	1455	3975	**2098**	**744**	3076	3955	6177	3638	5721	5100	5650	5264	2148	3073	3014	2908	**4144**	**1363**	**0.0001523**
**ICOS CD278**	30	39	42	45	225	111	118	170	77	74	93	155	**98**	**60**	104	114	160	120	437	464	426	468	78	96	72	80	**218**	**172**	**0.0325481**

Supplemental Table 5 Ihle

Supplemental Table 5. DSP Normalized Antibody Counts: Macrophage ROI.

Antigen	Lytic														Blastic													p-Value
	Patient 1				Patient 2				Patient 3				Average	ST DEV	Patient 1			Patient 2				Patient 3				Average	ST DEV	
**Pan-Cytokeratin**	13455	10690	4255	12025	687	3183	3659	3276	6862	3031	398	460	**5165**	**4565**	738	250	443	513	575	1917	1550	650	864	701	692	**808**	**492**	**0.0024587**
**CD3**	410	811	435	259	702	65	120	62	454	299	9059	5504	**1515**	**2808**	8440	4750	2428	3653	4830	3111	3082	2373	1218	540	495	**3175**	**2288**	**0.0686366**
**CD68**	20651	22289	41782	11686	28007	27933	12735	19832	22898	13659	164831	55504	**36817**	**42233**	26048	29016	8642	61411	74815	76434	54136	69489	46458	39367	42646	**48042**	**21688**	**0.2191454**
**P-AKT (Ser473)**	11569	15897	1715	4575	539	548	1341	625	3439	7128	369	1894	**4137**	**4993**	1451	1443	1425	533	534	565	592	456	400	747	314	**769**	**445**	**0.0186042**
**P-STAT3 (Tyr705)**	157	144	607	126	135	155	296	134	203	213	320	247	**228**	**136**	694	810	727	695	642	570	654	463	348	460	417	**589**	**148**	**0.0000023**
**Ki-67**	6800	5874	5001	2626	5521	1210	10920	1295	288	384	427	331	**3390**	**3413**	786	451	1542	2126	2967	1947	2038	240	269	259	293	**1174**	**979**	**0.0253627**
**Bcl-2**	417	457	620	409	790	1267	1654	1159	450	256	1049	539	**756**	**432**	1723	1401	1081	2024	1831	1595	1664	1098	955	1086	998	**1405**	**379**	**0.0005016**
**CD45**	1266	2305	3055	1144	1107	335	158	291	967	1153	4729	7141	**1971**	**2089**	7397	6120	1803	8050	7730	4866	5649	5940	2341	2322	1438	**4878**	**2491**	**0.0031009**
**CD4**	3605	4621	14198	1823	5400	2280	2020	1703	5989	5976	12467	12394	**6040**	**4507**	11056	7715	3392	7792	7336	5895	6147	10913	5259	5362	4752	**6874**	**2420**	**0.2955022**
**FoxP3**	69	87	158	47	30	45	88	53	73	43	155	99	**79**	**42**	223	372	237	158	160	182	131	120	104	144	88	**174**	**80**	**0.0007850**
**GZMB**	725	1017	831	648	636	993	1150	986	703	470	1321	875	**863**	**242**	2436	2131	3447	3915	3950	2624	2948	886	595	724	588	**2204**	**1320**	**0.0011645**
**CD44**	905	1700	4255	993	6163	1705	5439	1179	4599	4994	8467	9248	**4137**	**2901**	18016	11192	2645	12768	14847	11244	10139	7625	3241	3362	3426	**8955**	**5283**	**0.0060880**
**CD8A**	361	529	409	350	656	156	216	108	184	384	6234	8007	**1466**	**2673**	5945	4488	2087	4188	4258	3530	3815	1928	650	466	293	**2877**	**1895**	**0.0812364**
**CD45RO**	1056	1001	1715	648	516	179	241	158	554	427	3554	4031	**1173**	**1304**	3572	2211	701	796	876	825	828	2126	949	828	627	**1303**	**933**	**0.3938490**
**CD11c**	6249	5381	9857	5505	15150	10072	5351	5747	12604	7214	98776	76512	**21535**	**31399**	15788	9901	3257	9621	10698	10566	7397	63768	35004	42114	34124	**22022**	**19043**	**0.4824949**
**CD14**	682	832	1353	535	147	210	163	175	363	213	2233	1316	**685**	**651**	1063	2156	524	854	729	746	707	1639	882	1155	793	**1022**	**479**	**0.0874231**
**CD163**	413	462	693	312	852	571	283	521	655	384	9273	4723	**1595**	**2709**	1175	2101	865	2694	2553	2236	1658	646	446	391	417	**1380**	**898**	**0.4022317**
**CD66B**	4399	5386	1056	4817	96	27	68	28	1213	43	175	247	**1463**	**2101**	58	67	143	207	74	741	154	117	98	494	80	**203**	**217**	**0.0307800**
**CD56**	7088	5366	70845	2507	308	198	1023	135	295	9988	1952	317	**8335**	**19944**	1242	1084	12624	696	467	389	605	1063	263	305	402	**1740**	**3626**	**0.1465293**
**HLA-DR**	3483	5648	7330	1980	12586	1087	3369	806	11633	5933	39023	20999	**9490**	**10983**	57119	18147	6938	23723	20747	11758	14628	76697	42637	40723	30278	**31218**	**21309**	**0.0026255**
**B7-H3**	142173	165024	273846	64649	21718	27835	63046	20128	67884	52629	56442	77472	**86070**	**73800**	90044	66764	28421	22397	17567	21217	17307	25756	11097	20735	11580	**30262**	**24919**	**0.0133582**
**B7-H4 VTCN1**	105	128	165	60	53	114	155	72	97	85	253	158	**120**	**56**	383	420	490	259	217	205	232	141	134	178	161	**256**	**121**	**0.0010303**
**PD-L1**	443	539	798	297	403	336	657	310	341	213	952	687	**498**	**229**	1888	1553	1806	1313	1118	1243	1064	530	623	1040	754	**1176**	**448**	**0.0000725**
**PD1**	92	92	211	71	151	74	125	63	133	43	447	322	**152**	**121**	718	426	362	266	253	215	221	703	385	368	293	**383**	**176**	**0.0006712**
**STING TMEM173**	13517	18756	18961	28765	4811	8480	9918	8433	5714	2774	2961	3170	**10522**	**8072**	11483	11473	3150	27949	24027	15998	19840	7243	6388	5822	8434	**12891**	**8072**	**0.2447933**
**VISTA**	3001	4180	1861	1615	1229	1444	1218	1244	2364	598	3097	2671	**2043**	**1033**	1354	1778	1105	2927	3159	2979	3538	1116	1315	1310	1337	**1993**	**946**	**0.4517587**
**OX40L**	46	51	79	24	72	70	108	68	41	85	204	158	**84**	**51**	340	298	420	218	216	211	252	120	86	184	158	**227**	**97**	**0.0001005**
**Beta-2-Microglobulin**	57899	63237	66095	30738	11217	4974	9778	4092	23435	14384	18109	21746	**27142**	**22652**	43529	31056	12454	23488	19645	17098	16980	24760	14348	22160	14889	**21855**	**9001**	**0.2390058**
**IDO-1**	2155	2264	3398	2036	1483	2241	3424	1997	1876	1409	4408	3205	**2491**	**911**	9183	8848	10401	7337	7221	7714	6971	3521	3513	4557	3511	**6616**	**2474**	**0.0000117**
**ICOS CD278**	141	267	238	73	110	207	301	165	152	85	680	411	**236**	**170**	845	359	347	298	208	278	293	177	131	144	148	**293**	**200**	**0.2324160**

Supplemental Table 6 Ihle

Supplemental Table 6. DSP Normalized Antibody Counts: T Cell ROI.

Antigen	Lytic													Blastic														p-Value
	Patient 1				Patient 2				Patient 3			Average	ST DEV	Patient 1				Patient 2				Patient 3				Average	ST DEV	
**Pan-Cytokeratin**	2689	902	813	2270	656	1618	2092	665	404	766	442	**1211**	**809**	738	250	443	1557	1501	1049	3067	1720	238	185	640	181	**964**	**870**	**0.4886265**
**CD3**	2928	34437	21747	607	976	1437	533	18964	34021	9330	15378	**12760**	**13118**	8440	4750	2428	7529	4802	2830	2820	3210	17735	4968	5133	2166	**5568**	**4306**	**0.1084383**
**CD68**	34256	35154	20991	7650	8170	17411	10646	50262	29985	17118	22508	**23105**	**13208**	26048	29016	8642	10217	24791	41919	40716	43058	39795	32650	30518	10642	**28168**	**12636**	**0.3593421**
**P-AKT (Ser473)**	2490	459	2285	715	658	1110	950	419	928	931	651	**1054**	**694**	1451	1443	1425	1173	653	565	1272	701	592	619	488	596	**915**	**397**	**0.5675189**
**P-STAT3 (Tyr705)**	383	387	871	154	119	220	166	271	121	291	286	**297**	**213**	694	810	727	658	936	715	1731	648	565	453	363	825	**760**	**344**	**0.0009567**
**Ki-67**	738	1055	1530	7648	6540	19184	10265	937	1170	320	312	**4518**	**5971**	786	451	1542	1673	49072	29001	32675	3883	507	314	369	225	**10041**	**16864**	**0.3053658**
**Bcl-2**	815	1273	1123	936	733	1700	1302	961	767	912	1093	**1056**	**286**	1723	1401	1081	1805	2573	1792	2636	1757	2804	1607	1073	1641	**1824**	**570**	**0.0007278**
**CD45**	4968	141108	55112	270	355	338	206	10708	20542	4209	12594	**22764**	**42410**	7397	6120	1803	11432	8012	6458	6639	5765	40920	7453	7490	14521	**10334**	**10112**	**0.3635516**
**CD4**	6404	37184	32165	2797	2291	5248	2581	20972	23690	11541	26567	**15585**	**12937**	11056	7715	3392	8676	8485	7130	6968	7698	30315	11525	11571	8842	**10281**	**6706**	**0.2423489**
**FoxP3**	61	129	310	49	35	65	50	148	161	58	130	**109**	**81**	223	372	237	216	153	147	362	193	169	111	103	88	**198**	**92**	**0.0221655**
**GZMB**	571	1635	2440	925	763	2666	639	1035	605	863	1015	**1196**	**734**	2436	2131	3447	2589	3110	3072	4259	4818	2171	1196	1219	737	**2599**	**1230**	**0.0035109**
**CD44**	2390	17190	13885	1570	2334	3877	1540	11768	6538	8224	14624	**7631**	**5844**	18016	11192	2645	11417	5811	5960	5480	19511	12068	5176	7327	5312	**9160**	**5349**	**0.5213671**
**CD8A**	6598	27316	18823	514	818	2754	656	16635	24900	8380	11163	**10778**	**9832**	5945	4488	2087	10538	2610	2670	2449	3014	3734	2258	2038	1196	**3585**	**2527**	**0.0376913**
**CD45RO**	1447	16071	13923	265	347	499	281	5385	9403	2628	5412	**5060**	**5716**	3572	2211	701	4225	1305	1005	1025	959	7980	2553	2726	2758	**2585**	**2039**	**0.1984562**
**CD11c**	21381	39536	19578	2018	3249	2926	2524	60809	21268	18602	16835	**18975**	**18034**	15788	9901	3257	11411	2239	5597	6389	7013	49360	43851	34339	7518	**16389**	**16477**	**0.7240041**
**CD14**	760	636	1685	157	97	209	146	1565	848	1106	1665	**807**	**627**	1063	2156	524	658	348	358	767	945	1547	1030	1312	834	**962**	**521**	**0.5282061**
**CD163**	288	1128	1433	230	386	346	210	5446	3592	1338	3227	**1602**	**1741**	1175	2101	865	868	1493	1050	1261	2349	798	443	379	353	**1095**	**637**	**0.3786417**
**CD66B**	3698	60311	349	26	37	56	29	394	242	78	286	**5955**	**18060**	58	67	143	111	5601	2564	2116	920	63	148	347	238	**1031**	**1672**	**0.3885947**
**CD56**	18952	4866	2459	974	296	577	973	4744	484	1290	1405	**3365**	**5413**	1242	1084	12624	1294	3066	644	1117	439	993	429	558	397	**1991**	**3426**	**0.4812811**
**HLA-DR**	6476	18657	15531	1085	3550	4934	2165	27762	18201	12395	12594	**11213**	**8397**	57119	18147	6938	21313	2664	10255	9901	3085	56648	54804	28318	15385	**23715**	**20925**	**0.0760201**
**B7-H3**	134552	20042	61677	30968	23145	32856	34931	139917	48428	131367	129870	**71614**	**50731**	90044	66764	28421	60944	17344	26914	26897	36563	14376	10126	30166	14481	**35253**	**24658**	**0.0487829**
**B7-H4 VTCN1**	105	169	407	82	46	123	73	148	161	116	130	**142**	**96**	383	420	490	389	232	193	841	252	143	263	163	154	**327**	**199**	**0.0107671**
**PD-L1**	449	1184	2479	293	246	603	297	678	888	446	885	**768**	**639**	1888	1553	1806	1552	1166	1219	4535	1390	1056	1108	612	662	**1546**	**1021**	**0.0400246**
**PD1**	216	475	930	111	86	181	89	456	404	475	416	**349**	**251**	718	426	362	752	235	234	612	254	919	540	417	468	**495**	**220**	**0.1549712**
**STING TMEM173**	14250	107476	30112	7810	4183	12406	13159	3031	7224	5916	4007	**19052**	**30309**	11483	11473	3150	8334	38385	34714	26617	15443	13145	6358	8336	6098	**15294**	**11603**	**0.7058408**
**VISTA**	2207	10448	2982	560	706	1597	998	3228	6498	3171	2966	**3215**	**2917**	1354	1778	1105	1005	4184	4452	5575	2780	1880	1141	2759	852	**2405**	**1570**	**0.4261707**
**OX40L**	44	177	291	84	66	97	68	160	202	107	78	**125**	**75**	340	298	420	505	242	235	1323	342	232	180	152	181	**371**	**317**	**0.0225043**
**Beta-2-Microglobulin**	37750	59337	100077	7489	7069	14862	6488	49079	19008	38707	45901	**35069**	**28595**	43529	31056	12454	28841	20164	20144	20998	23328	63224	33019	28730	37001	**30207**	**13402**	**0.6149607**
**IDO-1**	2218	8370	6410	1716	1340	2737	1839	3265	2543	2803	4736	**3452**	**2190**	9183	8848	10401	7029	5984	5283	8430	6915	3111	7448	2867	4033	**6628**	**2437**	**0.0034851**
**ICOS CD278**	255	959	1065	116	98	194	107	1146	1816	640	729	**648**	**559**	845	359	347	915	302	302	606	357	1162	425	341	265	**519**	**296**	**0.5060462**

Supplemental Table 7. Antibody Panel for DSP

Antigen	Function
Pan-Cytokeratin	Signaling
AKT	Signaling
P-AKT	Signaling
STAT3	Signaling
STAT3 (Phospho Y705)	Signaling
Ki-67	Signaling
Bcl-2	Signaling
Beta-Catenin	Signaling
CD45	Immune Cell Profiling
CD3	Immune Cell Profiling
CD4	Immune Cell Profiling
FoxP3	Immune Cell Profiling
GZMB	Immune Cell Profiling
CD44	Immune Cell Profiling
CD8A	Immune Cell Profiling
CD45RO	Immune Cell Profiling
CD11c	Immune Cell Profiling
CD14	Immune Cell Profiling
CD68	Immune Cell Profiling
CD163	Immune Cell Profiling
CD66B	Immune Cell Profiling
CD56	Immune Cell Profiling
HLA-DR	Immune Cell Profiling
B7-H3	Immune Checkpoint
B7-HA VTCN1	Immune Checkpoint
PD-L1	Immune Checkpoint
PD1	Immune Checkpoint
STING TMEM173	Immune Checkpoint
VISTA	Immune Checkpoint
OX40L CD252 TXGP1	Immune Checkpoint
Beta-2-Microglobulin	Immune Checkpoint
IDO-1	Immune Checkpoint
ICOS CD278	Immune Checkpoint
